# Bovine viral diarrhea virus suppresses type I IFN production by inducing MAVS degradation via autophagy mediated by the ROS-endoplasmic reticulum stress axis

**DOI:** 10.1128/jvi.01643-25

**Published:** 2025-12-09

**Authors:** Jing Wang, Jiangfei Zhou, Yixin Wang, Wenlu Fan, Xinyue Xia, Jiarui Chen, Haiyue Zhu, Qianyao Wang, Xiao Li, Yimei Liu, Jiayi Xiang, Han Yu, Moxuan Mao, Renjie Xu, Jiacun Liu, Shuo Jia, Yuan Li, Yigang Xu

**Affiliations:** 1 Key Laboratory of Applied Technology on Green-Eco-Healthy Animal Husbandry of Zhejiang Province, College of Veterinary Medicine, Zhejiang A&F University 722545, Hangzhou, People's Republic of China; 2 College of Animal Science, Technology, Zhongkai University of Agriculture and Engineering 47894, Guangzhou, China; 3 Key Laboratory of Medical Molecular Virology of the Ministry of Education, School of Basic Medical Sciences, Fudan University 58305, Shanghai, China; 4 Key Laboratory for Animal Disease Control and Pharmaceutical Development of Heilongjiang Province, Northeast Agricultural University 12430, Harbin, People's Republic of China; University of Michigan Medical School, Ann Arbor, Michigan, USA

**Keywords:** bovine viral diarrhea virus (BVDV), ROS-ER stress-autophagy axis, mitochondrial antiviral signaling protein (MAVS), type I interferon

## Abstract

Bovine viral diarrhea virus (BVDV) is a major animal pathogen with a broad host range, causing gastrointestinal, respiratory, and reproductive diseases in cattle worldwide. BVDV exists as two biotypes: cytopathic (cp) and non-cytopathic (ncp). Although both cpBVDV and ncpBVDV have developed sophisticated strategies to evade or subvert host antiviral innate immune response, the underlying mechanisms remain incompletely understood. Autophagy, a process essential for maintaining cellular homeostasis, plays an important role in regulating viral replication and antiviral immunity. In this study, we demonstrated that the induction of autophagy with rapamycin enhanced the production of infectious progeny for both cpBVDV and ncpBVDV, whereas pharmacological inhibition of autophagy with 3-MA reduced viral yields. We further showed that modulating autophagy significantly influenced the early stages of the viral life cycle and the production of type I IFN (IFN-I). Notably, overexpression of BECN1 suppressed the synthesis of IFN-α and IFN-β, thereby promoting the replication of both cpBVDV and ncpBVDV. Conversely, RNA interference-mediated knockdown of BECN1 potentiated the antiviral innate immune response and restricted viral replication. Mechanistically, BECN1 was found to inhibit RIG-I–MAVS pathway activation by promoting ubiquitination and subsequent degradation of mitochondrial antiviral signaling (MAVS) protein, leading to suppression of IFN-I production. Additionally, both cpBVDV and ncpBVDV were shown to induce autophagy via the ROS-endoplasmic reticulum stress axis. These findings deepen our understanding of how BVDV evades host immunity and may inform the development of preventive strategies against BVDV infection.

**IMPORTANCE:**

Bovine viral diarrhea virus (BVDV), the causative agent of bovine viral diarrhea-mucosal disease, is a major global threat to cattle health. BVDV employs sophisticated strategies to evade host defense and facilitate its replication. Understanding these mechanisms is crucial for developing effective vaccines and antiviral agents. Our study elucidates how cytopathic BVDV and non-cytopathic BVDV subvert the host’s antiviral innate immune response by exploiting autophagy to inhibit the RIG-I–MAVS pathway. A key finding is that BECN1-mediated autophagy directly targets MAVS protein for degradation via a specific BECN1 and MAVS interaction. Furthermore, we demonstrate that BVDV activates autophagy through ROS-ER stress axis to promote its replication. These insights reveal a novel immune evasion mechanism of BVDV and highlight the therapeutic potential of autophagy inhibition in treating BVDV-related diseases.

## INTRODUCTION

Bovine viral diarrhea-mucosal disease (BVD-MD), caused by bovine viral diarrhea virus (BVDV), is an economically significant contagious disease in cattle, characterized clinically by diarrhea, fever, mucosal erosion, persistent infection (PI), and immunosuppression. BVDV ranks among the most prevalent and destructive pathogens in cattle herds worldwide, leading to substantial economic losses in the global livestock industry (1, 2). BVDV is categorized into two biotypes based on their cytopathic effects in cell culture: cytopathogenic (cp) BVDV (cpBVDV) and non-cytopathogenic (ncp) BVDV (ncpBVDV). The ncp biotype, which is the most frequently isolated from clinical cases, is primarily responsible for establishing PI. When cattle with PI are superinfected with antigenically related or homologous cpBVDV strain, they may develop fatal mucosal disease (3). A hallmark molecular distinction between the ncpBVDV and cpBVDV is the expression of the non-structural protein NS3 (also known as p80). This difference often arises during PIs, where ncpBVDV can acquire mutations in the NS2-3 genomic region, giving rise to cpBVDV variants that may trigger lethal disease. At the protein level, ncpBVDV produces primarily the uncleaved NS2-3 polyprotein (p125), whereas cpBVDV expresses both the full-length NS2-3 and significant amounts of the separate NS3 protein. In ncpBVDV, the NS2-3 region remains intact, preventing efficient cleavage. In contrast, cpBVDV frequently harbors insertions of cellular sequences within NS2-3, which create novel cleavage sites and lead to the constitutive processing of the polyprotein into discrete NS2 and NS3 proteins (4–6). Throughout prolonged virus-host co-evolution, both cpBVDV and ncpBVDV have evolved sophisticated strategies to evade or counteract the host’s antiviral innate immune response (7). Nevertheless, the precise molecular mechanisms underlying this immune subversion remain largely unexplored.

Cellular autophagy is a highly conserved intracellular process that degrades and recycles cellular components, playing an essential role in maintaining cellular homeostasis and modulating immune defenses. Autophagy is activated under various stress conditions, such as nutrient deprivation, reactive oxygen species (ROS) accumulation, endoplasmic reticulum (ER) stress, and pathogen infection, through the formation and lysosomal fusion of autophagosome (8). Accumulating evidence indicates that autophagy can eliminate invading viruses and support cell survival by facilitating nutrient recycling. However, several viruses have evolved strategies to hijack or suppress autophagic machinery to enhance viral replication and infectivity. Previous studies have demonstrated that BVDV infection exploits the autophagy pathway to promote viral propagation in host cells (7, 9). It is well established that cellular stress resulting from viral replication often acts as a trigger for autophagy induction (10, 11). Although BVDV infection has been reported to induce both ER stress and oxidative stress (12, 13), the precise mechanisms linking BVDV-induced stress to autophagy regulation remain poorly defined.

As the first line of defense against viral infection, interferons (IFNs) play a pivotal role in host antiviral immunity. The mitochondrial antiviral signaling (MAVS) protein functions as a critical switch in the innate immune signaling cascade triggered by most RNA viruses. Upon viral RNA recognition, retinoic acid-inducible gene I (RIG-I) and melanoma differentiation-associated protein 5 (MDA5) activate MAVS, leading to the formation of functional prion-like aggregates that subsequently promote the phosphorylation and nuclear translocation of IFN regulatory factor 3 (IRF3), thereby initiating IFN-mediated antiviral response (14). In previous work, we demonstrated that BVDV infection upregulates lncRNA-cylindromatosis (CYLD), which functions as a competing endogenous RNA (ceRNA) for miR-2383. This interaction enhances the expression of CYLD, thereby suppressing RIG-I-mediated type I IFN (IFN-α/β) production and facilitating BVDV replication (15). However, the molecular mechanisms by which BVDV evades host innate immunity are still not fully elucidated.

The interplay between autophagy and IFN response represents an important mechanism in virus-host interactions. Although autophagy can function as a cell-autonomous defense against viral infection, numerous viruses have evolved the ability to exploit the autophagic machinery to subvert host innate immunity. Accumulating evidence indicates that certain viruses activate autophagy to evade antiviral IFN signaling (16–18). A detailed understanding of the interaction between BVDV and host cellular pathways is therefore essential to elucidate the underlying mechanisms. In this study, we systematically investigated how autophagy induced by both biotypes of BVDV influences viral replication. We found that cpBVDV and ncpBVDV trigger autophagy via ROS-ER stress axis, which in turn suppresses RIG-I–MAVS pathway-mediated IFN-I production. This process was further associated with a functional interaction between MAVS and BECN1. These findings deepen our understanding of how BVDV evades innate immune defenses and offer valuable insights for the development of targeted antiviral strategies.

## RESULTS

### BVDV induces the formation of autophagosome and autophagic flux

**Fig 1:**
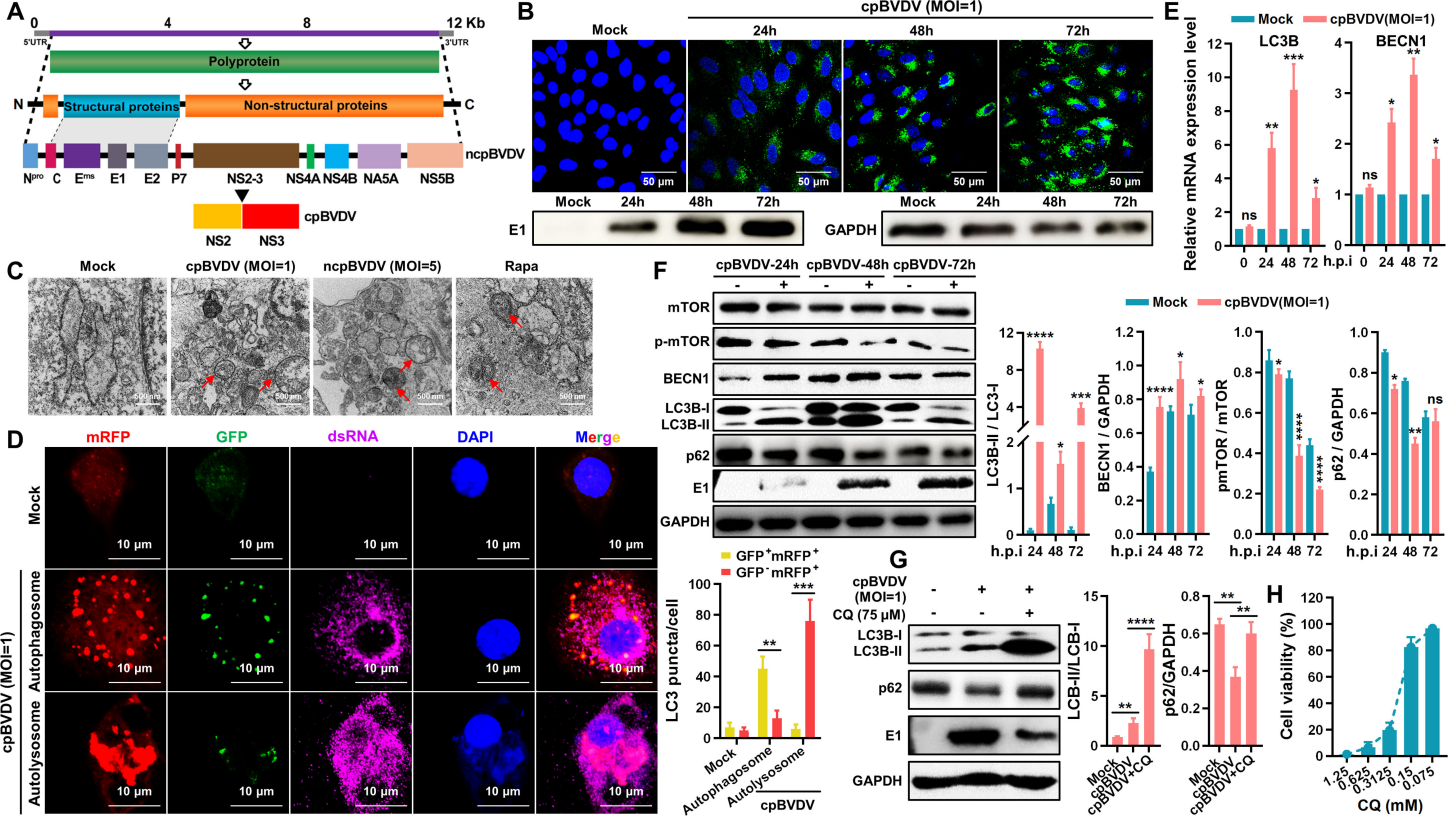
CpBVDV induces autophagy and complete autophagic flux in BT cells. (**A**) Genomic and proteomic structures of cpBVDV and ncpBVDV. (**B**) Detection of cpBVDV infection (MOI = 1) in BT cells by IFA and Western blot. (**C**) TEM images showing autophagosome formation in BT cells infected with cpBVDV (MOI = 1) or ncpBVDV (MOI = 5). Untreated cells and Rapa-treated cells served as negative and positive controls, respectively. (**D**) Autophagic flux assessed using adenoviral reporter Ad-mCherry-GFP-LC3 (MOI = 1) in cpBVDV-infected BT cells. (**E**) qRT-PCR analysis of LC3B and BECN1 mRNA expression in cpBVDV-infected BT cells at 0, 24, 48, and 72 h post-infection, using *β-actin* as an internal reference. (**F**) Western blot analysis of key autophagy-related proteins p-mTOR, BECN1, and LC3B in cpBVDV-infected BT cells (MOl = 1). (**G**) LC3B-II accumulation detected by Western blot in BT cells pretreated with CQ (0.075 mmol/L) and infected with cpBVDV (MOI = 1). (**H**) Cytotoxicity of CQ evaluated by CCK-8 assay. Data are presented as mean ± SD from three independent experiments. *, *P* < 0.05; **, *P* < 0.01; ***, *P* < 0.001; ****, *P* < 0.0001; ns, not significant.

**Fig 2:**
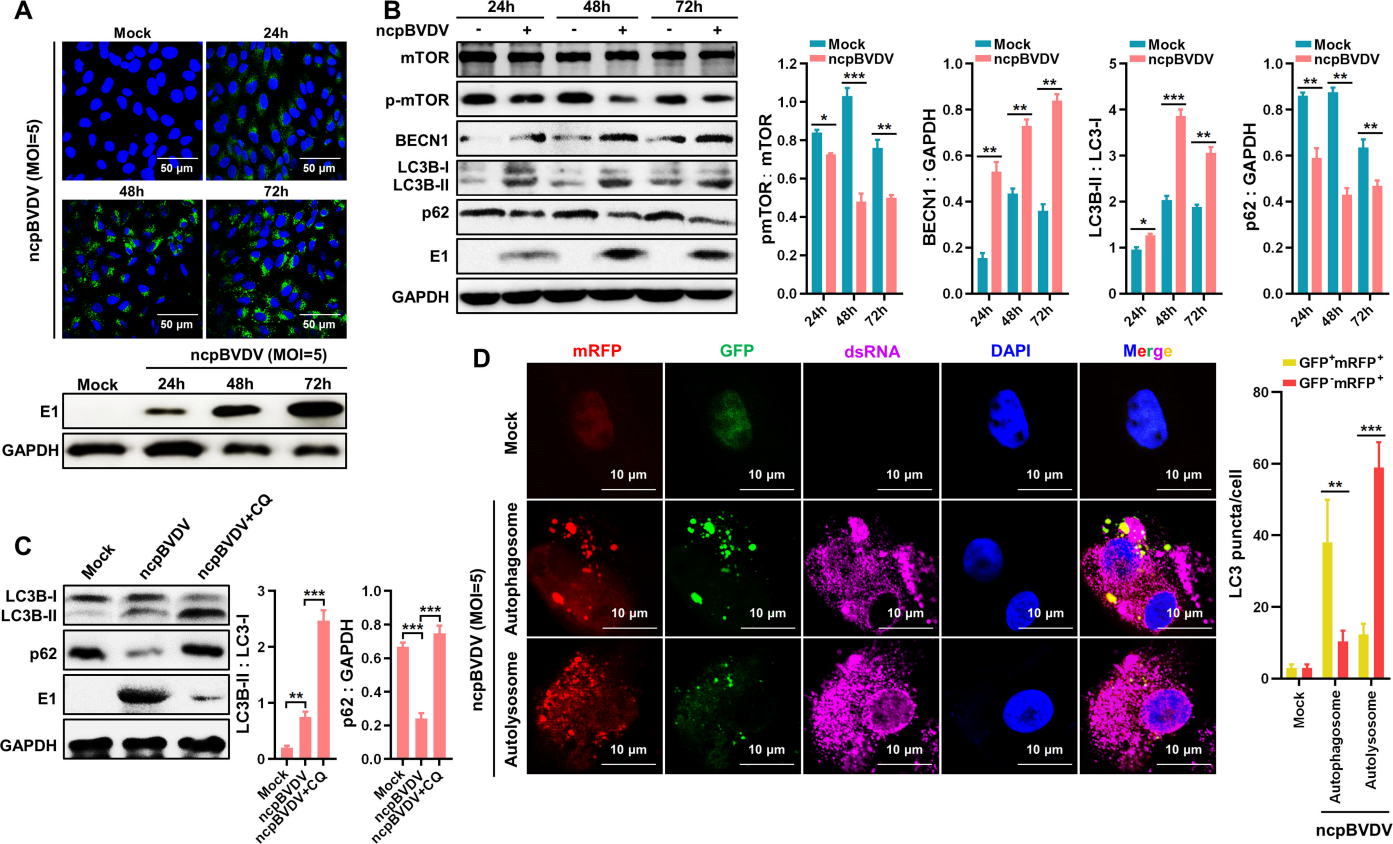
NcpBVDV infection induces complete autophagy progression in BT cells. (**A**) Detection of ncpBVDV infection (MOl = 5) in BT cells by IFA and Western blot. (**B**) Western blot analysis of key autophagy-related proteins p-mTOR, BECN1, and LC3B in ncpBVDV-infected BT cells (MOl = 5). GAPDH was used as an internal reference. (**C**) LC3B-II accumulation detected by Western blot in BT cells pretreated with CQ (0.075 mmol/L) and infected with ncpBVDV (MOI = 5). (**D**) Autophagic flux analyzed using the adenoviral reporter Ad-mCherry-GFP-LC3 (MOI = 1) in ncpBVDV-infected BT cells (MOl = 5). Data are presented as mean ± SD from three independent experiments. *, *P* < 0.05; **, *P* < 0.01; ***, *P* < 0.001.

The genomic and proteomic organizations of cpBVDV and ncpBVDV are schematically illustrated in Fig. 1A. Infection of BT cells with cpBVDV (multiplicity of infection [MOI] = 1, Fig. 1B) and ncpBVDV (MOI = 5, Fig. 2A) at the indicated time points was confirmed by immunofluorescence assay (IFA) and Western blot. Transmission electron microscopy (TEM) revealed the presence of autophagic vesicles in cpBVDV- and ncpBVDV-infected BT cells, exhibiting characteristic double-membrane structures measuring 500–1,000 nm in diameter (Fig. 1C). Similar structures were observed in rapamycin (Rapa)-treated positive control cells, displaying typical autophagic features with clearly discernible contents. In contrast, no significant autophagic vesicle-like structures were detected in mock-infected cells. To further validate the integrity of the autophagic process induced by cpBVDV/ncpBVDV—encompassing autophagosome formation, lysosomal fusion, and autolysosome maturation—we monitored autophagic flux using confocal microscopy in BT cells co-infected with cpBVDV/ncpBVDV and the adenoviral reporter Ad-mCherry-GFP-LC3. In this system, the acidic environment of autolysosomes quenched GFP fluorescence, while mRFP signal remained stable. Thus, autophagosomes appear as yellow puncta (GFP^+^mRFP^+^), whereas autolysosomes are identified as red puncta (GFP^−^mRFP^+^). As shown in Fig. 1D and 2D, infection with either cpBVDV or ncpBVDV induces yellow puncta formation, indicating autophagosome formation. The subsequent appearance of red-only puncta confirmed autophagosome-lysosome fusion and demonstrated that both biotypes of BVDV enhance autophagic flux. At the transcriptional level, cpBVDV infection significantly upregulated the mRNA expression of LC3B and BECN1 (Fig. 1E). Consistent with this, Western blot analysis showed increased protein levels of BECN1 and lipidated LC3B-II upon cpBVDV infection (Fig. 1F). A concurrent decrease in phosphorylated mammalian target of Rapa (p-mTOR) was observed at different time points post-infection (Fig. 1F). Similar trends were also seen in ncpBVDV-infected BT cells (Fig. 2B), suggesting that both biotypes induce autophagy via suppression of mTOR activation. Autophagic flux, reflecting the rate of autophagic degradation, was quantified by measuring LC3B-II accumulation in the presence of the lysosomal inhibitor chloroquine (CQ). Western blot analysis showed significant increases in LC3B-II levels in both cpBVDV- and ncpBVDV-infected BT cells after treatment with CQ (Fig. 1G and 2C), at working concentration of 0.075 mmol/L (Fig. 1H), indicating that BVDV infection promotes not only autophagosome formation but also their subsequent degradation.

### BVDV-induced autophagy inhibits IFN-I production

**Fig 3:**
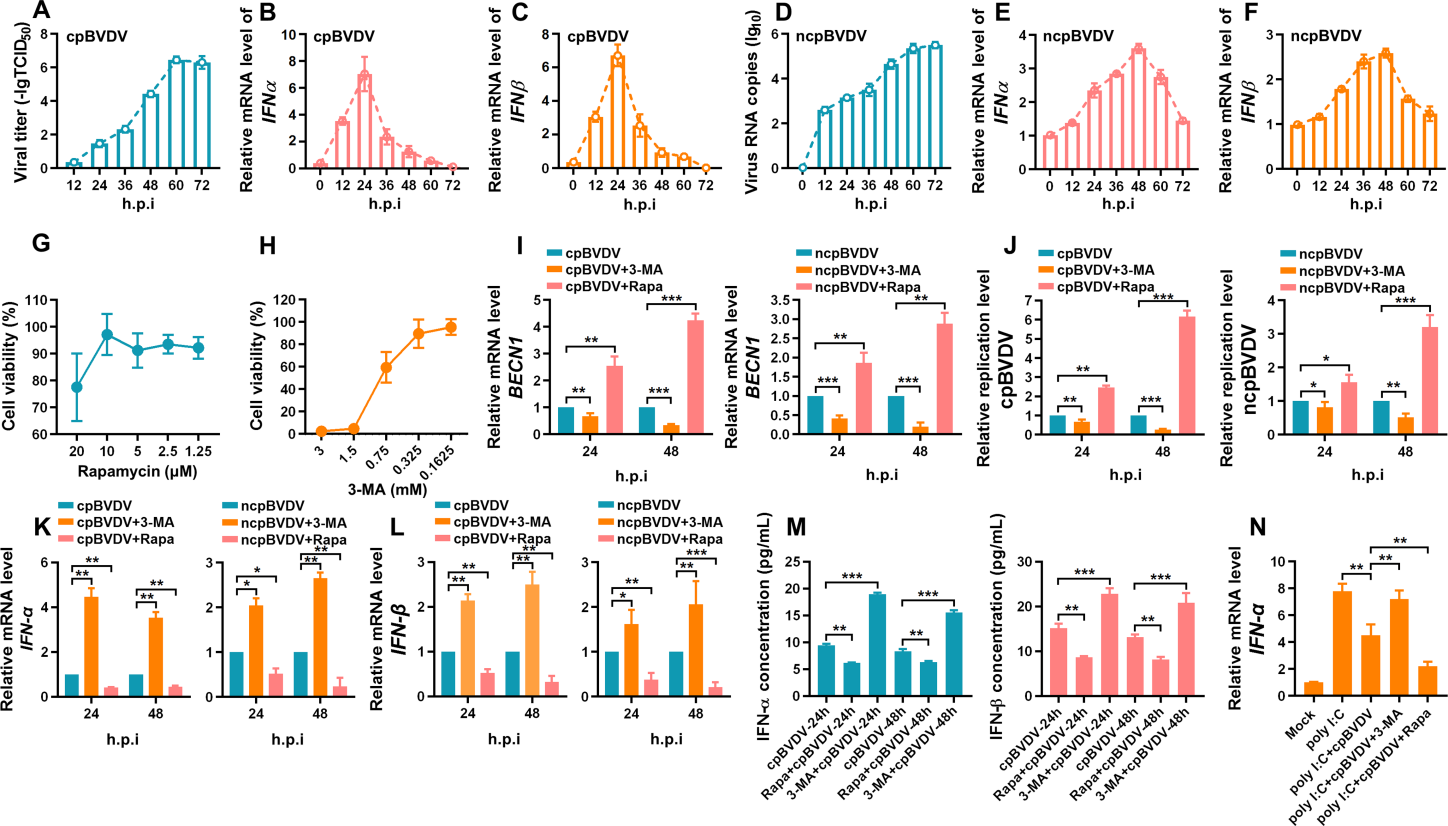
Inhibition of autophagy enhances IFN-I production in BVDV-infected cells. (**A**) Viral titers of cpBVDV (MOI = 1) at indicated time points post-infection, determined by TCID_50_ assay. (**B, C**) IFN-α (**B**) and IFN-β (**C**) mRNA expression in cpBVDV-infected BT cells (MOI = 1) at different time points, analyzed by qRT-PCR with *β-actin* as internal reference. (**D**) Viral RNA copies of ncpBVDV (MOI = 5) at different time points post-infection, measured by absolute quantification. (**E, F**) IFN-α (**E**) and IFN-β (**F**) mRNA expression in ncpBVDV-infected BT cells (MOI = 5), assessed by qRT-PCR normalized to *β-actin*. (**G, H**) Cytotoxicity of Rapa (**G**) and 3-MA (**H**) evaluated by CCK-8 assay. (**I**) BECN1 mRNA expression in BT cells pretreated with Rapa or 3-MA and infected with cpBVDV (MOI = 1) or ncpBVDV (MOI = 5), analyzed by qRT-PCR with *β-actin* as internal reference. (**J**) Viral replication levels of cpBVDV or ncpBVDV in BT cells pretreated with Rapa or 3-MA relative to virus alone group. (**K, L**) IFN-α (**K**) and IFN-β (**L**) mRNA expre ssion autophagy modulator-treated and BVDV-infected BT cells, measured by qRT-PCR using *β-actin* as an internal reference. (**M**) Secreted IFN-α/β concentrations in the supernatants of cpBVDV-infected BT cells pretreated with Rapa or 3-MA, quantified by ELISA. (**N**) IFN-α mRNA expression in poly(I:C)-pretreated cells with or without BVDV infection in the presence of Rapa or 3-MA, analyzed by qRT-PCR normalized to *β-actin*. Data are presented as mean ± SD from three independent experiments. *, *P* < 0.05; **, *P* < 0.01; ***, *P* < 0.001.

The interplay between cellular autophagy and viral infection is complex, with playing contradictory roles as either a host defense mechanism or a proviral factor. In this study, we investigated how autophagy influences BVDV infection using Rapa, an autophagy inducer by inhibiting the mTOR pathway (19), and 3-methyladenine (3-MA), an autophagy inhibitor by inhibiting the PI3K class III protein (20). Viral replication (Fig. 3A and D) and mRNA expression of IFN-α (Fig. 3B and E) and IFN-β (Fig. 3C and F) were measured in BT cells at different time points after cpBVDV or ncpBVDV infection. At the concentrations used—10 µM for Rapa and 0.325 mM for 3-MA, as determined by the CCK-8 assay (Fig. 3G and H), Rapa significantly increased BECN1 mRNA expression, while 3-MA suppressed it in BT cells infected with cpBVDV or ncpBVDV (Fig. 3I). Furthermore, we observed that Rapa significantly enhanced cpBVDV and ncpBVDV replication, an opposite effect exerted by 3-MA (Fig. 3J). Growing evidence suggests that virus-induced autophagy can modulate antiviral immune responses (21–23). Here, Rapa downregulated both mRNA (Fig. 3K and L) and protein levels (Fig. 3M) of IFN-α/β in BVDV-infected BT cells. Conversely, 3-MA enhanced IFN-α/β expression. We also pretreated BT cells with poly(I:C) and examined IFN-β production with or without BVDV infection in the presence of Rapa or 3-MA. As shown in Fig. 3N, IFN-α mRNA levels were significantly higher in the poly(I:C)+ BVDV + 3 MA group compared to poly(I:C)+BVDV group but markedly lower in the poly(I:C)+BVDV + Rapa group. These findings collectively demonstrate that autophagy benefits BVDV replication, consistent with previous report (24).

### BECN1 promotes BVDV replication by inhibiting type I IFN production

**Fig 4:**
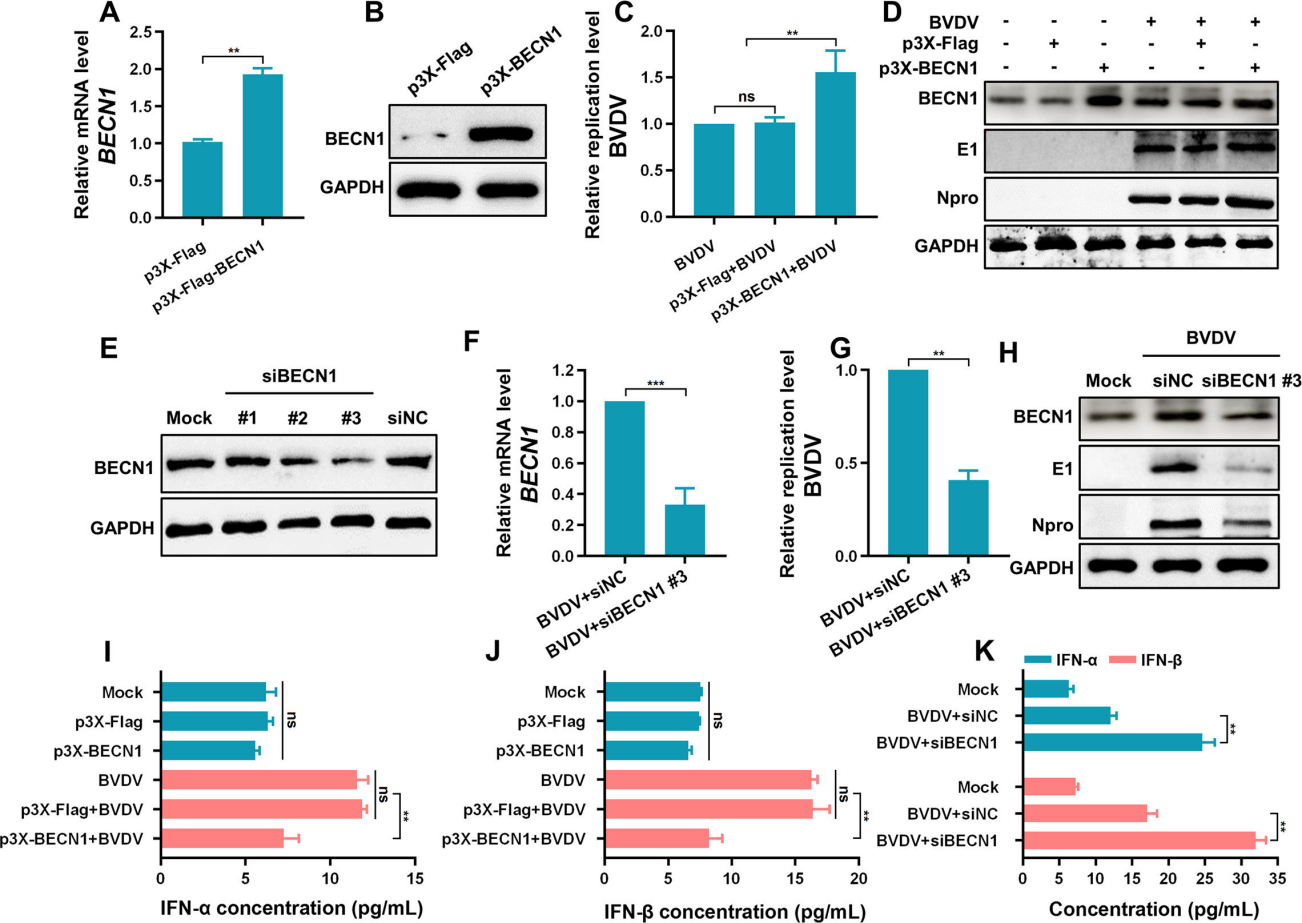
BECN1 facilitates BVDV replication by suppressing IFN-I production. (**A**) qRT-PCR analysis of BECN1 mRNA expression in BT cells transfected with p3×-BECN1 normalized to *β-actin*. (**B**) Western blot confirming BECN1 protein expression in p3×-BECN1-transfected BT cells. (**C**) CpBVDV (MOI = 1) replication level in BECN1-overexpressing BT cells, assessed by qRT-PCR. (**D**) CpBVDV viral protein expression in BECN1-overexpressing BT cells, analyzed by Western blot. (**E**) Knockdown efficiency of BECN1-targeting siRNAs evaluated by Western blot. (**F**) BECN1 mRNA expression in BT cells transfected with si*BECN1* #3 and infected with cpBVDV (MOI = 1), measured by qRT-PCR. (**G**) Viral replication level of cpBVDV (MOI = 1) in BECN1-knockdown BT cells relative to virus plus siNC group. (**H**) Viral protein levels of cpBVDV (MOI = 1) in BECN1-knockdown BT cells, analyzed by Western blot. (**I, J**) IFN-α (**I**) and IFN-β (**J**) levels in supernatant of BECN1-overexpressing BT cells after cpBVDV infection (MOI = 1), measured by ELISA. (**K**) IFN-α and IFN-β levels in the supernatants of BECN1-knockdown BT cells post cpBVDV infection (MOI = 1), determined by ELISA. Data are presented as mean ± SD from three independent experiments. **, *P* < 0.01; ***, *P* < 0.001; ns, not significant.

BECN1, a key regulator of autophagy, is involved in multiple stages of autophagosome formation, maturation, and transport. Previous studies have shown that ubiquitin-specific protease 19 suppresses IFN-I production by deubiquitinating BECN1, thereby disrupting RIG-I–MAVS interaction (25). Additionally, classical swine fever virus (CSFV)-induced autophagy has been reported to inhibit IFN-I signaling via BECN1-MAVS association, facilitating viral replication. Building on these findings and our own data, we hypothesize that BECN1 may contribute to the suppression of IFN-I production during BVDV-induced autophagy. To test this hypothesis, we constructed a plasmid p3×-Flag-BECN1 (p3×-BECN1) for exogenous BECN1 expression. Successful overexpression was confirmed at both mRNA (Fig. 4A) and protein levels (Fig. 4B). We found that BECN1 overexpression significantly enhanced cpBVDV replication (Fig. 4C) and viral protein expression (Fig. 4D). We also designed small interfering RNA (siRNA) targeting *BECN1* (siBECN1), among which si*BECN1* #3 showed the highest knockdown efficiency (Fig. 4E). Knockdown of *BECN1* in BVDV-infected BT cells (Fig. 4F) led to markedly reduced viral replication (Fig. 4G) and viral protein expression (Fig. 4H), supporting the notion that BECN1 promotes efficient viral replication. Furthermore, BECN1 overexpression significantly suppressed the production of IFN-α (Fig. 4I) and IFN-β (Fig. 4J) in BVDV-infected BT cells compared with infection alone (*P* < 0.01). Conversely, BECN1 knockdown enhanced IFN-α and IFN-β production (Fig. 4K). Taken together, these results suggest that the autophagy-related protein BECN1 is involved in the inhibition of IFN-I production during BVDV infection.

### BVDV-induced autophagy suppressing IFN-I production is related to the BECN1 and MAVS interaction

**Fig 5:**
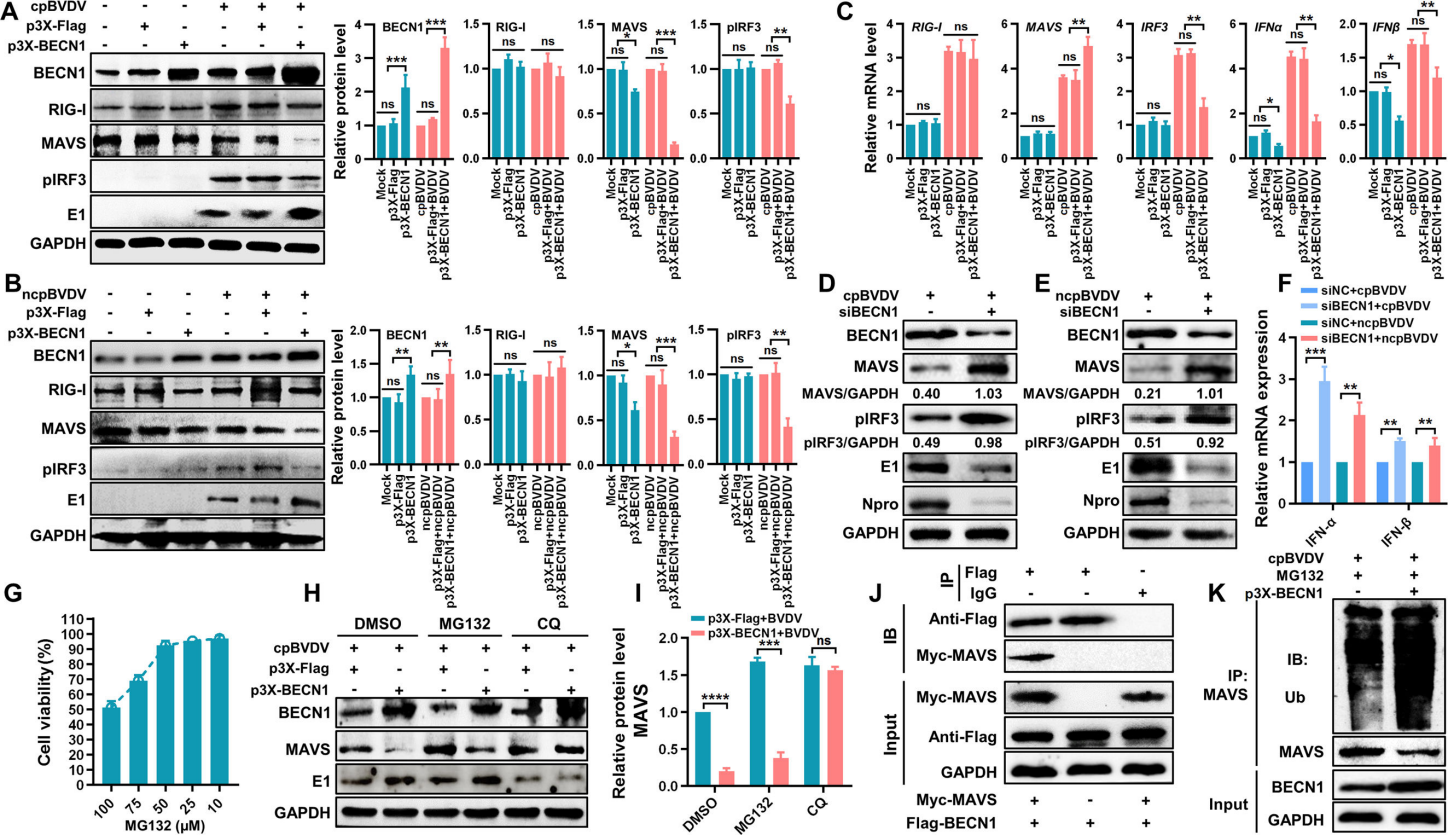
BVDV-induced autophagy suppresses IFN-I production through BECN1-MAVS interaction. (**A, B**) Western blot analysis of BECN1, RIG-I, MAVS, and pIRF3 in BECN1-overexpressing BT cells infected with cpBVDV (**A**) and ncpBVDV (**B**), using GAPDH as an internal control. (**C**) qRT-PCR analysis of RIG-I, MAVS, IRF3, IFN-α, and IFN-β mRNA expression in BECN1-overexpressing BT cells infected with cpBVDV (MOI = 1), normalized to *β-actin*. (**D, E**) Protein levels of BECN1, MAVS, pIRF3, and viral proteins (E1 and Npro) in BECN1-knockdown BT cells infected with cpBVDV (**D**) or ncpBVDV (**E**), assessed by Western blot. (**F**) IFN-α and IFN-β mRNA expression in BECN1-knockdown BT cells infected with cpBVDV (MOI = 1) or ncpBVDV (MOI = 5), determined by qRT-PCR using *β-actin* as the internal control. (**G**) Cytotoxicity of the proteasome inhibitor MG132 evaluated by CCK-8 assay. (**H**) MAVS protein expression in cpBVDV-infected BT cells with or without BECN1 overexpression and treated with CQ (0.075 mM) or MG132 (50 µM), analyzed by Western blot. (**I**) Grayscale quantification of MAVS protein levels shown in panel H. (**J**) Interaction between BECN1 and MAVS detected by Co-IP. (**K**) MAVS ubiquitination level in BECN1-overexpressing BT cells treated with MG132 and infected with cpBVDV, evaluated by Western blot. Data are presented as mean ± SD from three independent experiments. *, *P* < 0.05; **, *P* < 0.01; ***, *P* < 0.001; ns, not significant.

RIG-I acts as a primary sensor for RNA viruses by recognizing double-stranded RNA (dsRNA), 5′-triphosphorylated RNAs, and other pathogen-associated molecular patterns. This recognition triggers downstream signaling via MAVS, leading to IFN-I production. To elucidate whether BVDV-induced upregulation of BECN1 influences the RIG-I-like receptor (RLR) signaling pathway, we examined the expression of key proteins in the RIG-I pathway in BT cells with either overexpression or knockdown of BECN1 after cp/ncpBVDV infection. As shown in Fig. 5A and B, RIG-I protein levels remained unchanged (*P* > 0.05) in cp/ncpBVDV-infected BT cells regardless of BECN1 overexpression. In contrast, MAVS protein expression and IRF3 phosphorylation (pIRF3) were significantly reduced (*P* < 0.001 and *P* < 0.01, respectively) in BECN1-overexpressing cells infected with cp/ncpBVDV. At the mRNA level, BECN1 overexpression did not affect RIG-I expression (*P* > 0.05) but significantly downregulated *IRF3*, *IFN-α*, and *IFN-β* transcripts (*P* < 0.01) and upregulated MAVS expression (*P* < 0.01) (Fig. 5C). These results suggest that BECN1 promotes MAVS protein degradation without altering its transcription.

Following siRNA-mediated BECN1 knockdown, we observed a substantial increase in MAVS and pIRF3 levels (*P* < 0.001), along with significant suppression of viral replication in both cpBVDV- (MOI = 1, Fig. 5D) and ncpBVDV-infected (MOI = 5, Fig. 5E) BT cells. Moreover, IFN-α and IFN-β mRNA levels were significantly elevated in BECN1-knockdown BT cells after BVDV infection (Fig. 5F), indicating that BECN1 knockdown enhances RLR signaling and antiviral responses. To explore the mechanism of BVDV-induced MAVS degradation, BECN1-expressing BT cells infected with cpBVDV (MOI = 1, 24 h) were treated with DMSO, CQ (0.075 mmol/L, a lysosomal inhibitor), or MG-132 (50 µmol/L, a proteasome inhibitor, Fig. 5G) for 4 h. Western blot analysis revealed that CQ treatment significantly restored MAVS protein levels (Fig. 5H and I). Additionally, we found that MG-132 treatment also contributed to the inhibition of MAVS degradation. These findings suggest that both the autophagic-lysosomal and proteasomal pathways contribute to MAVS degradation, with the former playing a dominant role during BVDV infection. Furthermore, co-immunoprecipitation (Co-IP) assay confirmed an interaction between BECN1 and MAVS (Fig. 5J), and BECN1 was found to promote MAVS ubiquitination and subsequent degradation (Fig. 5K).

### BVDV-induced ER stress triggers autophagy

**Fig 6:**
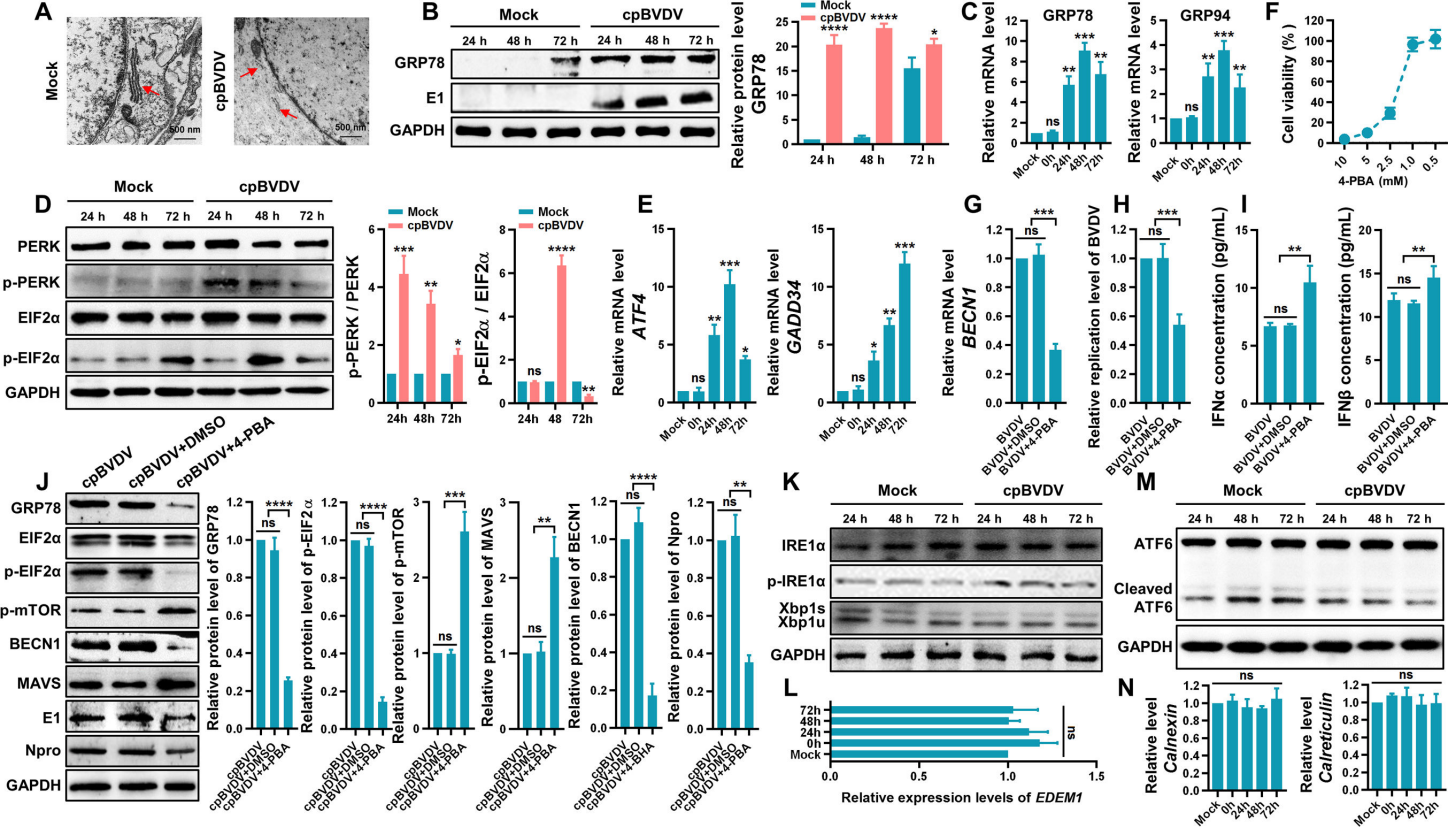
BVDV induces autophagy through ER stress to suppress IFN-I production. (**A**) TEM images showing ER swelling and rupture in cpBVDV-infected BT cells compared to mock-infected cells. (**B**) GRP78 protein expression in BT cells at 24, 48, and 72 h after cpBVDV infection, analyzed by Western blot. (**C**) ER chaperones GRP78 and GRP94 mRNA levels in cpBVDV-infected BT cells at indicated time points, measured by qRT-PCR, normalized to β*-actin*. (**D**) Protein levels of PERK, p-PERK, EIF2α, and p-EIF2α in cpBVDV-infected BT cells, assessed by Western blot using GAPDH as loading control. (**E**) PERK effectors ATF4 and GADD34 mRNA expression in cpBVDV-infected BT cells determined by qRT-PCR, normalized to *β-actin*. (**F**) Cytotoxicity of ER stress inhibitor 4-PBA evaluated by CCK-8 assay. (**G–I**) BECN1 mRNA levels (**G**), viral replication (**H**), and IFN-I (IFN-α/IFN-β) levels in 4-PBA (1 mM)-pretreated BT cells infected with cpBVDV (MOI = 1) relative to virus alone and virus plus DMSO groups. (**J**) Protein levels of GRP78, EIF2α, p-EIF2α, p-mTOR, BECN1, MAVS, and viral proteins (E1 and Npro) in 4-PBA-treated and cpBVDV-infected BT cells, detected by Western blot (GAPDH as control). (**K**) Protein levels of IRE1α, p-IRE1α, Xbp1s, and Xbp1u in BT cells infected with cpBVDV analyzed by Western blot. (**L**) EDEM1 (downstream of IRE1) mRNA expression in cpBVDV-infected BT cells, quantified by qRT-PCR (*β-actin* as the internal reference). (**M**) Protein levels of full-length and cleaved ATF6 in cpBVDV-infected BT cells at 24, 48, and 72 hpi. (**N**) Calreticulin and calnexin (ATF6 downstream regulators) mRNA expression in cpBVDV-infected BT cells determined by qRT-PCR. Data are presented as mean ± SD from three independent experiments. *, *P* < 0.05; **, *P* < 0.01; ***, *P* < 0.001; ****, *P* < 0.0001; ns, not significant.

The ER, a key organelle in eukaryotic cells, undergoes morphological changes under ES stress conditions. In this study, TEM revealed that the ER in cpBVDV-infected BT cells frequently displayed swelling and rupture, features absent in mock-infected cells (Fig. 6A). To further investigate ER stress induced by BVDV infection, we evaluated the expression of GRP78 (glucose-regulated protein, 78 kDa), an ER-resident chaperone and central regulator of the unfolded protein response (UPR) under both physiological and stress conditions, at different time points after cpBVDV infection. GRP78 protein levels were markedly elevated in infected BT cells (Fig. 6B). Moreover, quantitative real-time PCR (qRT-PCR) analysis showed significant upregulation of GRP94 mRNA, another major ER chaperone, consistent with the trend observed for GRP78 mRNA (Fig. 6C). These results indicate that cpBVDV infection triggers ER stress.

**Fig 7:**
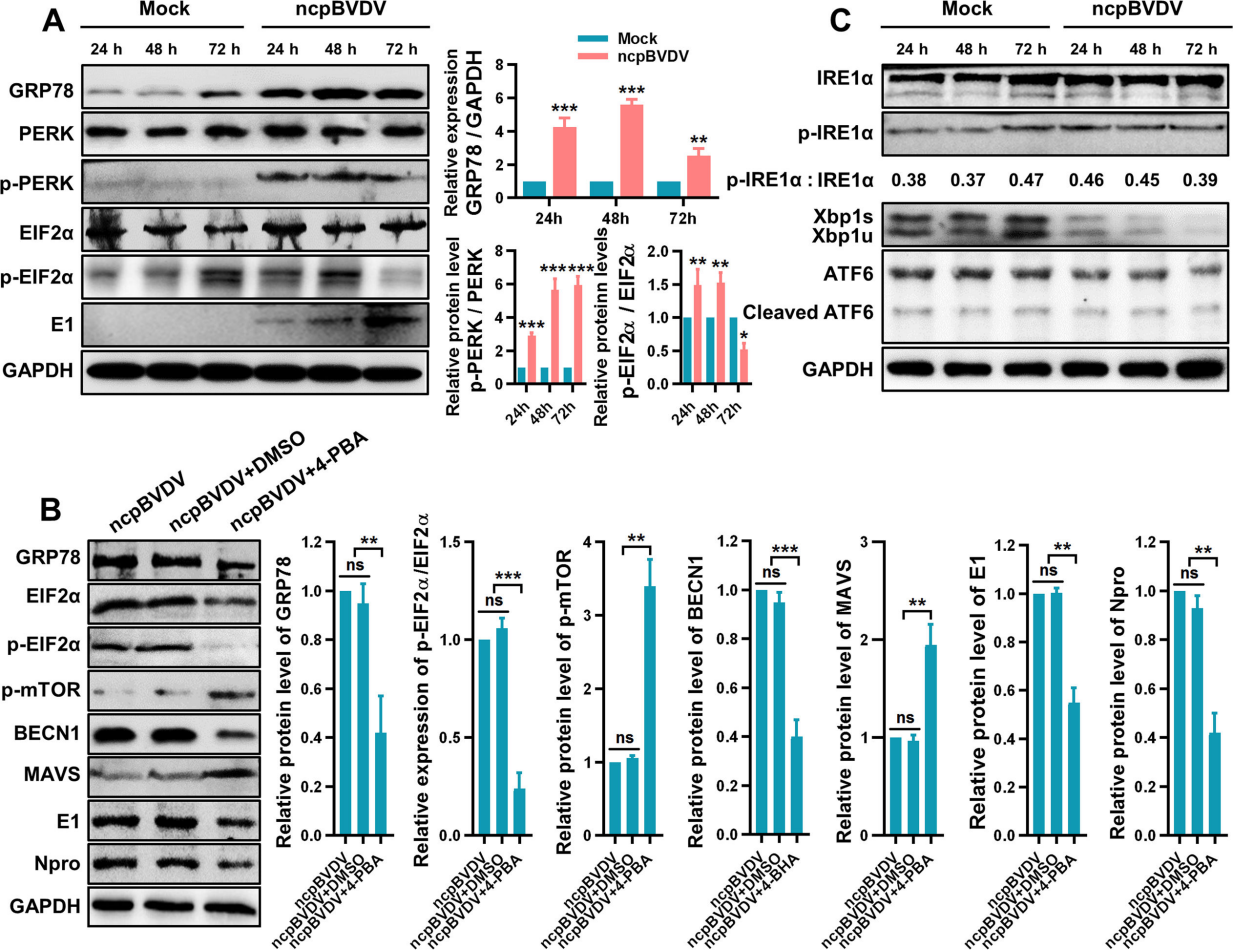
NcpBVDV activates the PERK branch of the UPR response to induce autophagy. (**A**) Western blot analysis of PERK, p-PERK, EIF2α, and p-EIF2α protein levels in ncpBVDV-infected BT cells at 24, 48, and 72 hpi. GAPDH was used as a loading control. (**B**) Protein expression of GRP78, EIF2α, p-EIF2α, p-mTOR, BECN1, MAVS, and viral proteins (E1 and Npro) in BT cells pretreated with 4-PBA and infected with ncpBVDV, detected by Western blot using GAPDH as a reference. (**C**) Activation status of the IRE1 branch (IRE1α, p-IRE1α, Xbp1s, and Xbp1u) and ATF6 branch (full-length and cleaved ATF6) of the UPR in ncpBVDV-infected BT cells, assessed by Western blot with GAPDH as control. Data are presented as mean ± SD from three independent experiments. *, *P* < 0.05; **, *P* < 0.01; ***, *P* < 0.001; ns, not significant.

The UPR is a conserved signaling network that aims to restore ER homeostasis upon stress and is mediated by three ER transmembrane sensors: protein kinase R-like ER kinase (PERK), activating transcription factor 6 (ATF6), and inositol-requiring enzyme 1 (IRE1) (26). Under ER stress, ATF6 translocates to the Golgi apparatus, where it is cleaved to release its N-terminus, which acts as a transcription factor in the nucleus (27, 28). PERK and IRE1 are activated through oligomerization and trans-autophosphorylation (29). Phosphorylated PERK (p-PERK) phosphorylates eukaryotic translation initiation factor 2α (EIF2α), attenuating global protein synthesis and promoting expression of activating transcription factor 4 (ATF4). Activated IRE1, possessing endonuclease activity, excises a 26-nucleotide intron from X-box binding protein-1 (XBP1u) mRNA, generating the spliced form XBP1s (30, 31). In cpBVDV-infected BT cells, we detected significant increases in p-PERK and phosphorylated EIF2α (p-EIF2α) levels (Fig. 6D). Concurrently, expression of ATF4 and GADD34, downstream effectors of the PERK pathway, was markedly upregulated (Fig. 6E). Pretreatment of BT cells with the ER stress inhibitor 4-phenylbutyric acid (4-PBA; 1.0 mmol/L, Fig. 6F) prior to cpBVDV infection (MOI = 1) significantly suppressed BECN1 mRNA expression compared to infection alone or infection with DMSO control (*P* < 0.001; Fig. 6G). Notably, 4-PBA treatment also reduced cpBVDV replication (*P* < 0.001; Fig. 6H) and enhanced IFN-I (IFN-α/IFN-β) production (*P* < 0.01; Fig. 6I). Furthermore, 4-PBA treatment strongly inhibited GRP78 expression and EIF2α phosphorylation (*P* < 0.0001; Fig. 6J), confirming effective suppression of cpBVDV-induced ER stress. Western bolt analysis also revealed upregulation of p-mTOR (*P* < 0.001), downregulation of BECN1 protein (*P* < 0.0001), and increased MAVS protein levels (*P* < 0.01) (Fig. 6J), suggesting that ER stress inhibition attenuates cpBVDV-induced autophagy, thereby preventing MAVS degradation and restraining viral replication. We also evaluated the activation status of the other two UPR branches. No significant changes were observed in IRE1 protein levels or phosphorylation, or in the ratio of spliced to unspliced Xbp1 (Xbp1s/Xbp1u), in cpBVDV-infected BT cells relative to mock-infected controls (Fig. 6K). Similarly, mRNA expression of EDEM1, an IRE1 downstream target, remained unchanged (*P* > 0.05; Fig. 6L). For the ATF6 pathway, no significant alterations were detected in full-length or cleaved ATF6 protein levels (Fig. 6M), or in the mRNA expression of its downstream targets calreticulin and calnexin proteins, which are downstream regulators of ATF6 (*P* > 0.05; Fig. 6N). Comparable results were observed in ncpBVDV-infected BT cells (Fig. 7A through C). Taken together, these data indicate that both biotypes of BVDV infection activate ER stress predominantly through the PERK signaling pathway.

### ROS-ER stress-autophagy axis benefits BVDV replication via promoting MAVS degradation

**Fig 8:**
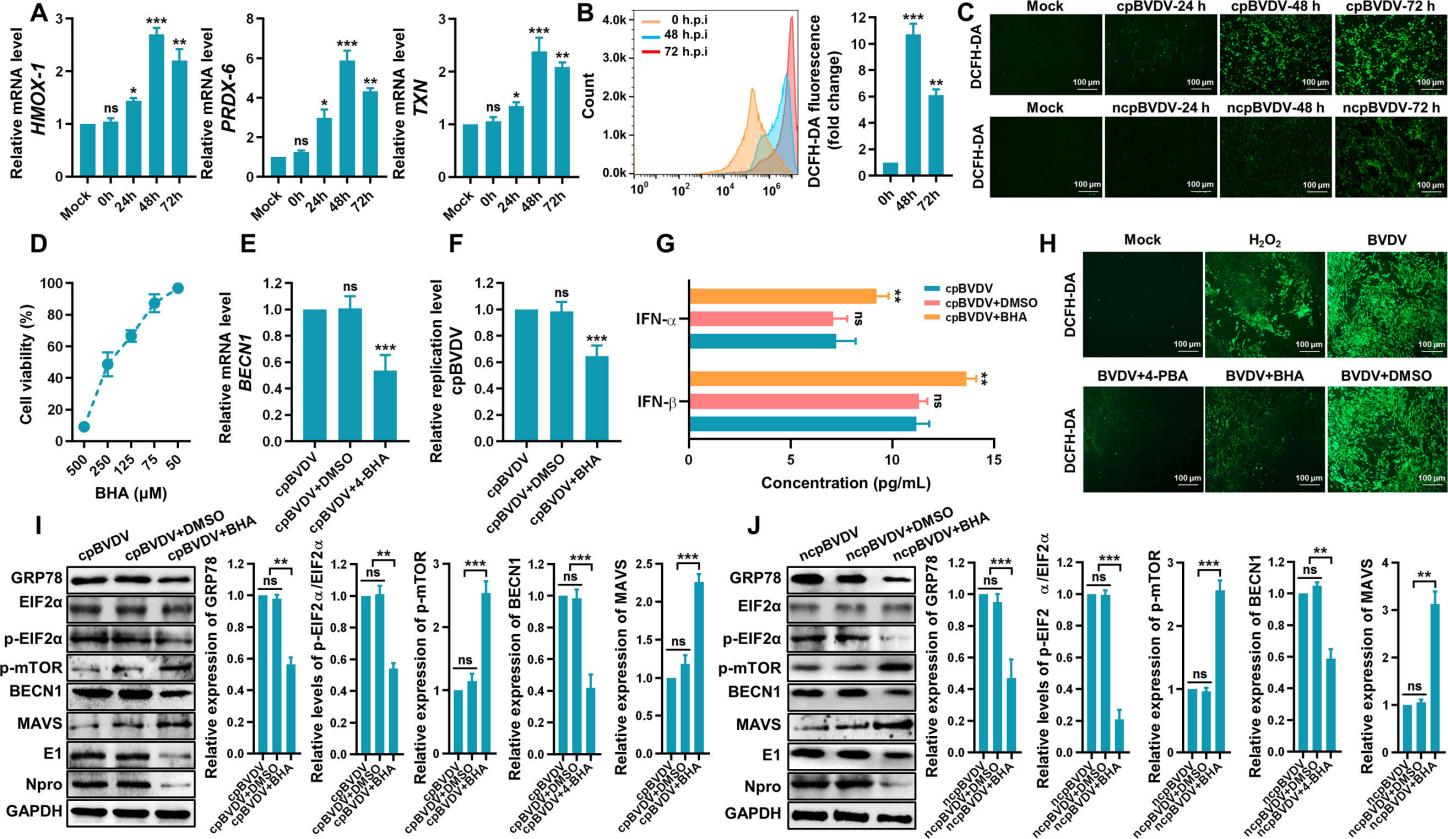
ER stress-mediated autophagy is associated with ROS accumulation during BVDV infection. (**A**) mRNA expression of oxidative stress-related gene *HMOX-1*, *PRDX-6*, and *TXN* in cpBVDV-infected BT cells, measured by qRT-PCR (normalized to *β-actin*). (**B**) ROS accumulation in cpBVDV-infected BT cells at various time points post-infection, measured by flow cytometry using the DCFH-DA probe. (**C**) Fluorescence microscope images showing ROS accumulation in BT cells infected with cpBVDV or ncpBVDV. (**D**) Cytotoxicity of the antioxidant BHA evaluated by CCK-8 assay. (E–G) BECN1 mRNA expression (**E**), viral replication (**F**), and IFN-I (IFN-α/IFN-β) levels (**G**) in BT cells pretreated with BHA (50 µM) and infected with cpBVDV (MOI = 1) relative to virus alone and virus plus DMSO groups. (**H**) Fluorescence microscopy detection of ROS (DCFH-DA staining) in cpBVDV-infected BT cells pretreated with 4-PBA or BHA. (**I, J**) Western blot analysis of GRP78, EIF2α, p-EIF2α, p-mTOR, BECN1, MAVS, and viral proteins (E1 and Npro) in BT cells pretreated with BHA and infected with cpBVDV at MOI = 1 (**I**) or ncpBVDV at MOI = 5 (**J**). Data are presented as mean ± SD from three independent experiments. *, *P* < 0.05; **, *P* < 0.01; ***, *P* < 0.001; ns, not significant.

ROS function as crucial signaling molecules in regulating diverse physiological and pathophysiological processes, including oxidative stress and apoptosis. Previous studies have reported that cpBVDV significantly induces ROS production, while ncpBVDV does not (32). However, other evidence suggests that ncpBVDV infection can also lead to ROS accumulation (33). In the present study, we observed a marked upregulation in mRNA expression of oxidative stress-related genes *HMOX-1*, *TXN*, and *PRDX-6* in cpBVDV-infected BT cells (Fig. 8A), indicating the induction of oxidative stress following cpBVDV infection. Using the DCFH-DA probe combined with flow cytometry, we assessed ROS accumulation in cpBVDV-infected BT cells (Fig. 8B). Fluorescence microscopy further confirmed ROS accumulation in both cpBVDV- and ncpBVDV-infected BT cells at various time points post-infection (Fig. 8C). Pretreatment with the antioxidant butylated hydroxyanisole (BHA; 50 µM, Fig. 8D) prior to cpBVDV infection (MOI = 1) significantly suppressed *BECN1* expression (*P* < 0.001; Fig. 8E) and reduced viral replication (*P* < 0.01; Fig. 8F), while enhancing IFN-I (IFN-α/IFN-β) production (*P* < 0.01; Fig. 8G). Moreover, treatment with the ER stress inhibitor 4-PBA substantially attenuated cpBVDV-induced ROS accumulation (Fig. 8H), suggesting that alleviating ER stress effectively induces oxidative stress. Importantly, antioxidant treatment with BHA in cpBVDV- or ncpBVDV-infected BT cells significantly decreased protein levels of GRP78 (*P* < 0.01; *P* < 0.001), p-EIF2α (*P* < 0.01; *P* < 0.001), and BECN1 (*P* < 0.001; *P* < 0.01), while increasing p-mTOR and MAVS levels (*P* < 0.001; *P* < 0.01), compared to virus-only and virus-plus-DMSO groups (Fig. 8I and J). These results imply that the ROS-ER stress-autophagy axis facilitates BVDV replication by promoting MAVS degradation, thereby suppressing RIG-I–MAVS-mediated IFN-I production and contributing to viral immune evasion.

## DISCUSSION

Autophagy is a critical biological process in viral pathogenesis, known for its complex and sometimes paradoxical roles in regulating inflammation, innate immunity, apoptosis, and cellular homeostasis (34). Our previous work showed that the expression of BVDV infection modulates key autophagy-related proteins, notably downregulating mTOR while upregulating ATG5 and BECN1. Concomitantly, we observed a significant suppression of proteins within the RIG-I–MAVS signaling pathway (35). Although autophagy generally supports immune system development and facilitates antimicrobial innate and adaptive responses, many intracellular pathogens have evolved strategies to evade or exploit this process to enhance their own survival and replication (36). However, the potential link between BVDV-induced autophagy and the suppression of IFN-I production has remained unclear. In this study, we therefore investigated the mechanisms through which autophagy induced by both cpBVDV and ncpBVDV antagonizes the host’s IFN-I-mediated antiviral innate immunity.

Our findings demonstrate that infection with either cpBVDV or ncpBVDV markedly enhances cellular autophagic activity, as evidenced by significant upregulation of the autophagy-related proteins BECN1 and LC3, which promotes autophagosome formation, consistent with our recent report (37). The complete autophagy process involves autophagosome formation, fusion with lysosomes, autolysosome generation, and subsequent degradation. In contrast, incomplete autophagy results from impaired autophagic lysosomal degradation, leading to accumulation of autophagic vesicles (38). Utilizing RFP-GFP-LC3 labeling systems, we observed that both cpBVDV and ncpBVDV infection induce complete autophagic flux in cells, consistent with earlier reports (33, 39–41). This differs from hepatitis C virus (HCV), another *Flaviviridae* member, which induces incomplete autophagy by blocking autophagosome-lysosome fusion, thereby preventing viral particle degradation and facilitating viral replication (42). In this study, we established that autophagy promotes the replication of both BVDV biotypes; however, the molecular mechanisms and biological implications of BVDV-induced complete autophagic flux require further investigation. It is well-known that the mTOR complex-1 acts as a central regulatory of autophagy (43). Multiple cellular signals and stressors converge on the mTOR pathway to initiate autophagy through suppression of mTOR activity. Our data showed that phosphorylation of mTOR is significantly inhibited in cpBVDV- and ncpBVDV-infected cells. Nevertheless, the precise mechanisms by which BVDV infection regulates mTOR phosphorylation to induce autophagy remain to be elucidated.

BVDV and other members of the *Flaviviridae* family are ER-tropic viruses known to disrupt ER homeostasis upon infection. Since cellular stress plays a critical role in autophagy regulation (44) and prolonged ER stress has been shown to trigger autophagy through IRE1α, PERK, and ATF6 signaling pathways (45), we investigated the involvement of ER stress in BVDV-induced autophagy. In this study, expression levels of the key ER chaperones GRP78 and GRP94 were significantly upregulated in BVDV-infected cells, suggesting that cpBVDV and ncpBVDV infection induces ER stress primarily through the PERK pathway, rather than via IRE1 or ATF6. This observation aligns with previous reports demonstrating PERK-mediated unfolded protein response activation during BVDV infection (46, 47). Furthermore, treatment with 4-PBA (an ER stress inhibitor) markedly attenuated autophagy activation induced by cpBVDV/ncpBVDV infection and significantly suppressed viral replication of both biotypes, indicating that ER stress-mediated autophagy facilitates BVDV propagation. Notably, 4-PBA also reduced the accumulation of ROS in infected cells. ROS serve as important signaling molecules involved in various physiological and pathophysiological processes and have been linked to oxidative stress, ER stress, and autophagy (48, 49). Previous studies indicate that cpBVDV-induced ROS promotes viral replication, whereas antioxidant treatment suppresses its replication (50). Although ncpBVDV has been reported not to induce ROS production in one study (32), our data show that it does elicit ROS production, consistent with findings by Li et al. (33). Thus, to explore the role of ROS in BVDV infection-induced ER stress-mediated autophagy-dependent viral replication is of considerable interest. In this work, we observed that antioxidant BHA treatment reduced BVDV-induced ROS accumulation, which in turn significantly inhibited both cpBVDV- and ncpBVDV-induced ER stress and autophagy, thereby suppressing viral replication.

There is growing evidence that autophagy contributes to viral pathogenesis (51). Although BVDV has evolved sophisticated strategies to evade or subvert host antiviral innate immunity, the mechanisms by which BVDV-induced autophagy suppresses IFN-mediated immune responses remain incompletely understood. BECN1, a key regulator of autophagy, is indispensable for autophagosome formation, maturation, and transport. Recent studies have shown that CSFV, which belongs to the same *Pestivirus* genus as BVDV, activates autophagy to suppress RIG-I–MAVS pathway-dependent type I IFN production via BECN1 and MAVS interaction (52). Similarly, early infection with dengue virus (DENV), another member of the *Flaviviridae* family, induces the ATG5-ATG12 conjugate, which attenuates MAVS-mediated IFN-stimulated gene expression, thereby helping the virus evade antiviral response prior to IFN signaling activation (53). In this study, we examined how modulating BECN1 expression—through overexpression and siRNA knockdown—affects the RLR-MAVS pathway and antiviral immunity. We found that BECN1 promotes proteolytic degradation of MAVS without affecting its transcription and disrupted RIG-I–MAVS interaction, thereby inhibiting RIG-I-mediated antiviral signaling and facilitating cp/ncpBVDV replication. Co-IP confirmed the BECN1-MAVS interaction, consistent with prior reports (52). These results align with findings by Li et al. (33) indicating that ncpBVDV induces mitophagy to suppress MAVS-driven innate immunity. We further demonstrated that BVDV promotes MAVS degradation partially through the ubiquitin-proteasome pathway, corroborating a previous study in which BVDV upregulates DNA-damage-inducible transcript 3 to enhance MAVS ubiquitination and degradation (54). MAVS degradation is a common viral immune evasion strategy. For example, the H1N1 nucleoprotein triggers mitophagy to degrade MAVS and dampen host innate immunity (55), and the hepatitis B virus X protein promotes MAVS ubiquitination at Lys136 to inhibit IFN-I production (56). Viruses can also cleave MAVS directly: proteases such as enterovirus 71 2Apro (57), coxsackievirus B 3C (58), HCV NS3-4A (59), and hepatitis A virus 3ABC (60) specifically cleave MAVS to suppress downstream signaling. MAVS function depends on its mitochondrial localization (61, 62). Notably, our study revealed that BECN1 enhances MAVS ubiquitination, although the precise molecular mechanisms warrant further investigation.

**Fig 9:**
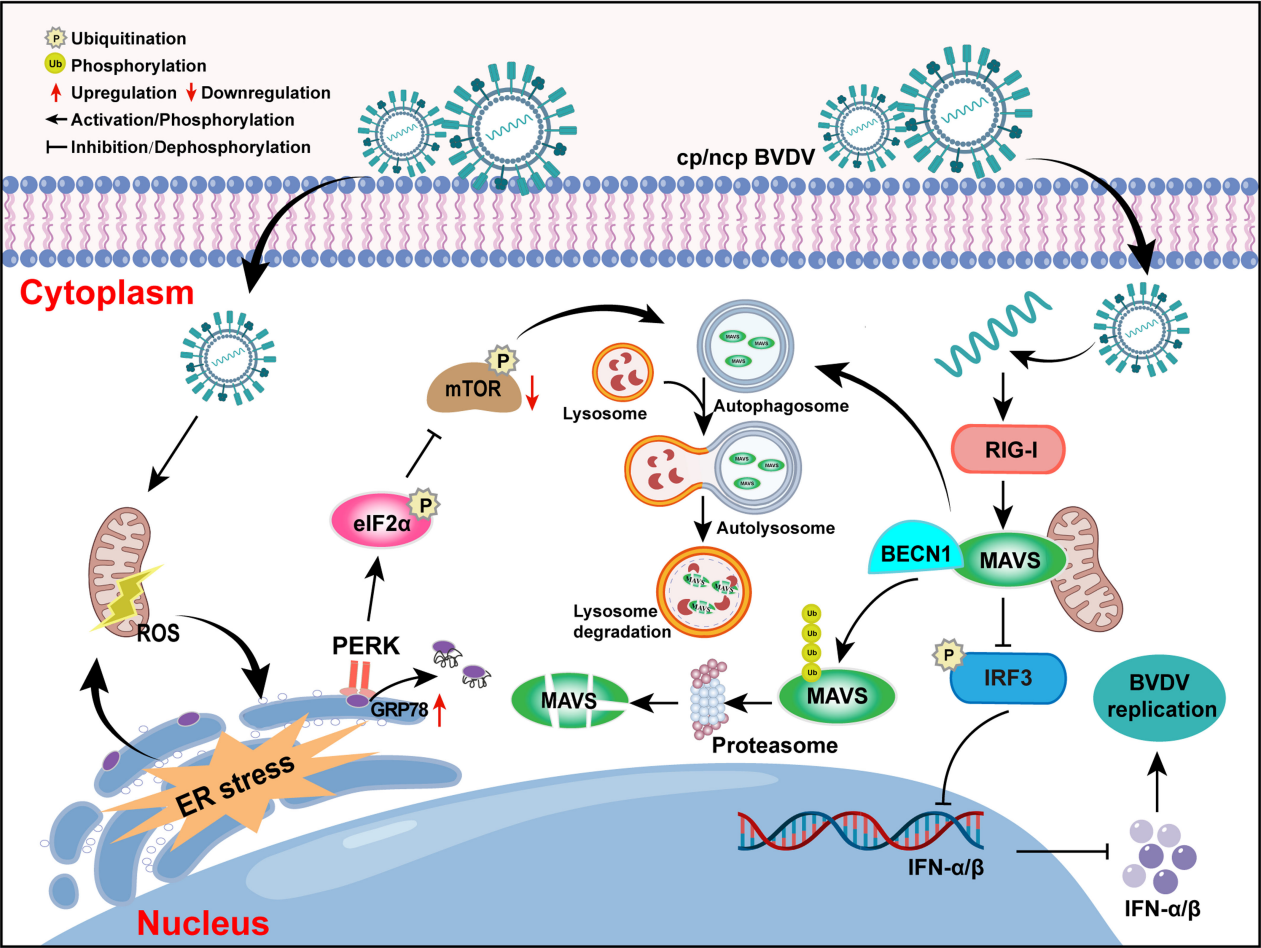
Proposed mechanism by which BVDV inhibits IFN-I signaling via BECN1-mediated autophagic degradation of MAVS.

In conclusion, this study preliminarily elucidates the mechanism by which cpBVDV/ncpBVDV infection-induced autophagy selectively degrades MAVS via BECN1–MAVS interaction, thereby suppressing the RIG-I–MAVS signaling pathway and impairing antiviral innate immunity. We further demonstrate that ROS-ER stress-autophagy axis promotes BVDV replication. As summarized in Fig. 9, BVDV targets BECN1-mediated autophagic degradation of MAVS to inhibit IFN-I signaling. These findings provide new insights into the immune evasion strategies shared by both biotypes of BVDV. However, the detailed mechanisms through which BVDV-induced autophagy enhances viral replication remain to be fully uncovered. Additionally, it should be clarified that cpBVDV AV69 and ncpBVDV BJ175170 are not a matched virus pair but rather separate isolates. Although we lack a matched non-cytopathic counterpart for cpBVDV AV69, we consider it methodologically valid to conduct key experiments in parallel with ncpBVDV BJ175170. The concordant results obtained from these experiments further bolster the reliability of our conclusions.

## MATERIALS AND METHODS

### Virus and cells

The cpBVDV strain AV69 (GenBank: KC695814.1) and ncpBVDV strain BJ175170 (GenBank: PX445908.1) used in this study, both belonging to genotype 1, were kept in our laboratory. BT cells were cultured in Dulbecco’s modified Eagle’s medium (DMEM; Gibco, USA) supplemented with 10% fetal bovine serum (FBS; Tianhang, China). Both cpBVDV and ncpBVDV infection in BT cells was detected via IFA. In this work, to ensure comparable viral replication dynamics between cpBVDV AV69 and ncpBVDV BJ175170 in subsequent experiments, we characterized replication kinetics of cpBVDV AV69 and ncpBVDV BJ175170 in BT cells using IFA and Western blot. When infected at a MOI of 1 for cpBVDV AV69 and MOI of 5 for ncpBVDV BJ175170, the two viruses exhibited similar replication levels in BT cells (Fig. 1B and 2A). Therefore, to minimize the potential influence of disparities in viral replication on host responses in follow-up experiments, cpBVDV at MOI = 1 and ncpBVDV at MOI = 5 were used to infect in all subsequent assays. This adjustment ensured that any observed differences in host responses could be attributed to strain-specific characteristics rather than variations in viral load.

### Determination of viral replication

The replication levels of cpBVDV and ncpBVDV were evaluated using different methods. For cpBVDV AV69, viral titers were determined by the 50% tissue culture infective dose (TCID_50_) assay as previously described (59). Briefly, confluent BT cell monolayers in 96-well plates were inoculated with serial 10-fold dilutions of the virus (eight replicates per dilution) for 2 h at 37°C; DMEM alone served as a mock control. After viral adsorption, cells were washed three times with phosphate-buffered saline (PBS) and maintained in DMEM with 2% FBS for 3–5 days. Cytopathic effect was monitored daily, and the TCID_50_ was calculated using the Reed-Muench method. For ncpBVDV BJ175170, replication level was determined by absolute quantification of viral RNA copies at various time points post-infection. Briefly, total RNA was extracted from infected BT cells using TRIzol reagent at the indicated time points and reverse-transcribed into cDNA. A standard curve was generated from a serially diluted plasmid containing BVDV 5′UTR to enable absolute quantification. Quantitative PCR (qPCR) was performed using Taq Pro Universal SYBR qPCR Master Mix (Vazyme, China) on an Applied Biosystems 7500 RT-PCR system.

### Transmission electron microscopy

Confluent monolayers of BT cells (80%–90% confluency in six-well plates) were infected with cpBVDV (MOI = 1) or ncpBVDV (MOI = 5), using Rapa-treated cells as a positive control. At 48-h post-infection, the cells were washed twice with ice-cold PBS (0.01 M, pH 7.4), harvested using a cell scraper, and washed again with PBS before fixation in 2.5% glutaraldehyde at 4°C for 24 h. After additional PBS washes, the samples were post-fixed in 1% osmium tetroxide for 60 min, dehydrated through a graded ethanol series, and embedded. Ultrathin sections were stained with 2% uranyl acetate and lead citrate (each for 5 min). Cellular ultrastructure features, including autophagosome-like vesicles and ER morphology, were examined by TEM.

### Quantitative real-time PCR

Total cellular RNA was extracted from the variously treated cell samples using the FastPure Cell/Tissue Total RNA Isolation Kit V2 (Vazyme, China) in accordance with the manufacturer’s protocol. First-strand cDNA was then synthesized with the HiScript III All-in-one RT SuperMix Perfect for qPCR (Vazyme, China). qPCR was performed on an Applied Biosystems 7500 RT-PCR system using Taq Pro Universal SYBR qPCR Master Mix (Vazyme, China). The relative mRNA expression levels of the target genes were normalized to *β-actin* as an internal control and calculated via the 2^−△△Ct^ method. All experiments were performed in triplicate. The qRT-PCR primer sequences are listed in Table 1.

**TABLE 1:** Primers used in this study^*a*^

Gene	Primer sequence (5′ to 3′)	Accession ID	Reference
*LC3B*	F: GCAGGCCACCGTTCACTCTTG	NM_001001169.1	This work
	R: GCAGCAGGAAGAGCAGATTGGG		
*BECN1*	F: ACTGGACACGAGCTTCAAGATTCTG	NM_001033627.2	This work
	R: CCTCCTGGGTCTCTCCTGGTTTC		
*GRP78*	F: CGGAGGAGGAGGACAAGAAGGAG	NM_001075148.1	This work
	R: ATAAGACGGCGTGATGCGGTTG		
*GRP94*	F: CGCAGGAACAGACGAGGAAGAAC	NM_174700.2	This work
	R: CACATTCCCTCTCCACACAGCATC		
*PRDX-6*	F: TGGCAAGAAATACCTCCGCTACAC	NM_174643.1	This work
	R: CATCCTCTGGCTCATGGTGCTAAG		
*HMOX-1*	F: CCGCTACCTGGGAGACCTGTC	NM_001014912.1	This work
	R: ACTTGGTGGCACTGGCGATATTG		
*TXN*	F: ACTGTCAGGTCGCTCGTCAGAC	NM_173968.3	This work
	R: CTCCTGCACTGTTCAAGGCTTCC		
*ATF4*	F: CCCAAACCCTACGACCCTCCTG	NM_001034342.2	This work
	R: TCCTGTTCCGCCCTCTTCTTCTG		
*GADD34*	F: ACCAGGAGAAGACACGGAGGAAG	NM_001046178.2	This work
	R: CAGGCACTCAGGAAGGCACTTG		
*EDEM1*	F: GCACGCCTTCTACTACGCCATC	NM_001103092.2	This work
	R: TGGTTGCCTGGTAGAGGAGATACG		
*ATF6*	F: GAGGAGCAAGACACATCGGATGAC	XM_024989877.1	This work
	R: GACAGGGAGGCGGAGGAATATAGAG		
*ERp57*	F: GCTCCTTGGTGTGGTCACTGTAAG	NM_174333.3	This work
	R: TCGTTGGCTGTAGCATCCATCTTG		
*Calnexin*	F: GATGAGCCTGAGTATGTGCCTGAC	NM_001105612.1	This work
	R: AGCCTCCCATTCTCCGTCCATATC		
*Calreticulin*	F: CAGAGGCTACATGAGGAGGAGGAG	NM_174000.2	This work
	R: CCAGCGGCAGCATCTTCTTCC		
*MAVS*	F: TGGCAGGCTGGTATCTAGGATGG	XM_025000838.1	This work
	R: CAAGGAGTTACTGTGGCTGATGGC		
*RIG-I*	F: CGTGGCAGAACAAATCAGACAATGG	XM_024996055.1	This work
	R: GGCGACCGAGGTAGCAATTAGAATC		
*IRF3*	F: CGGAAGGAAGTGTTGCGTTTAG	XM_024996055.1	This work
	R: GAGGTATTGTGTCTGCCATTGCT		
*IFN-α*	F: GTGCCCATTGTGTCCTGTCTGAG	NM_001017411.1	This work
	R: CGTGGTGCTGAAGAGCTGGAAG		
*IFN-β*	F: GGTAGCCCTGTGCCTGATTTCATC	NM_174350.1	This work
	R: AAGGCTCTGACGTTGTTGGAATCG		
*β-actin*	F: GCCAACCGTGAGAAGATGAC	AY141970.1	This work
	R: AGGCATACAGGGACAGCACA		
BVDV *5’UTR*	F: GGTAGCAACAGTGGTGAC	KF501393.1	This work
	R: GTAGCAATACAGTGGGCC		

^*a*^ Melting curve analysis for these primer pairs designed in this study was performed to confirm the specificity of qRT-PCRs.

### RNA interference

siBECN1 was designed and synthesized by GenePharma Co., Ltd. (Suzhou, China). A non-targeting siRNA was used as the negative control (siNC). BT cells were cultured to 80% confluence and transfected with siBECN1 using the jetPRIME *in vitro* DNA and siRNA transfection reagent (Polyplus, France). At 24-h post-transfection, the cells were infected with cpBVDV (MOI=1) or ncpBVDV (MOI=5). After transfection and infection, cell samples and supernatants were collected for further analysis. The knockdown efficiency of siBECN1 was assessed by qRT-PCR and Western blot. Cell viability was measured with Enhanced Cell Counting Kit-8 (CCK-8) (Beyotime, China) according to the manufacturer’s instructions.

### Overexpression

Recombinant eukaryotic plasmids expressing the host proteins BECN1 and MAVS were constructed by cloning the respective coding sequences amplified from the bovine cell genome into the vector p3×-Flag and pCMV-Myc using homologous recombination. The resulting constructs were designated as p3×-BECN1 (expressing Flag-tagged BECN1) and pCMV-MAVS (expressing Myc-tagged MAVS). After transfection with these plasmids using jetPRIME *in vitro* DNA and siRNA transfection reagent (Polyplus, France), cells and corresponding supernatants were collected for subsequent analysis.

### Western blot

Cells subjected to various treatments in six-well plates were harvested and lysed using RIPA Lysis Buffer (P0013B, Beyotime, China) supplemented with a protease and phosphatase inhibitor cocktail (Bimake, USA). Total protein concentration was determined with a Bicinchoninic Acid Protein Assay Kit (#P0011, Beyotime, China). Protein samples were separated by SDS-PAGE, transferred onto polyvinylidene difluoride membranes, and blocked with 5% skimmed milk at 37°C for 2 h. The membranes were incubated overnight at 4°C with the following primary antibodies (all diluted at 1:1,000 unless specified): ATF6 rabbit antibody (#A0202, ABclonal, China), phospho-mTOR-S2448 rabbit antibody (#AP0115, ABclonal, China), MAVS rabbit antibody (#A25005, ABclonal, China), BiP/GRP78 rabbit antibody (#A0241, ABclonal, China), phospho-IRE1-S724 rabbit antibody (#AP1146, ABclonal, China), PERK rabbit antibody (#AP1146, Abclonal, China), XBP1 rabbit antibody (#A1731, ABclonal, China), IRF3 rabbit antibody (#ab68481, Abcam, UK), BECN1 rabbit antibody (#AF5128, Affinity Biosciences, USA), SQSTM1/p62 (#A11483, ABclonal, China), LC3B rabbit antibody (#AF5640, Affinity Biosciences, USA), RIG-I rabbit antibody (#3743, CST, USA), eIF2α (D7D3) rabbit antibody (#9079, CST, USA; 1:1,000), phospho-eIF2α (Ser51) (D9G8) rabbit antibody (#3398, CST, USA; 1:1000), anti-BVDV E1 and Npro protein mouse polyclonal antibody (prepared in-house; 1:1,000), and either anti-GAPDH mouse (#GB15002-100) or rabbit (#GB15004-100) antibody (Servicebio, China, 1:3,000). After primary antibody incubation, the membranes were probed with appropriate horseradish peroxidase-conjugated secondary antibodies: Peroxidase AffiniPure Goat anti-Mouse IgG (H+L) (AB_10015289, Jackson ImmunoResearch, USA, 1:1,000) or Peroxidase AffiniPure Goat anti-Rabbit IgG (H+L) (AB_2307391, Jackson ImmunoResearch, USA, 1:1,000). Protein bands were visualized using Pierce ECL Western Blotting Substrate (#32209, Thermo Scientific, USA).

### Co-immunoprecipitation

To further examine the interaction between the autophagy protein BECN1 and the host protein MAVS, a Co-IP assay was conducted. Briefly, BT cells were grown in six-well plates until reaching over 80% confluency and then co-transfected with plasmids expressing Flag-BECN1 and Myc-MAVS. After transfection, the cells were incubated at 37°C with 5% CO_2_ for 36 h. Subsequently, the cells were harvested by scraping and lysed in IP lysis buffer supplemented with protease inhibitor (Beyotime, China). The lysates were centrifuged, and the supernatants were collected, precleared with anti-HA-agarose beads (Sigma, USA), and then incubated with the indicated antibodies. The beads were washed three times with PBS, followed by Western blot analysis.

### Enzyme-linked immunosorbent assay

To evaluate type I IFN production, supernatants from differently treated cells were collected. Levels of bovine IFN-α and IFN-β were then measured using specific enzyme-linked immunosorbent assay (ELISA) kits (MM-3472501 for IFN-α, MM-3694801 for IFN-β; Meimian, China) following the manufacturer’s protocols.

### Detection of ROS

To assess intracellular ROS levels, two loading methods for the DCFH-DA probe were employed. For the *in situ* loading, BT cells that were cultured in six-well plates (to ~70% confluence) and infected with BVDV were incubated at different time points post-infection with 1 mL of 10 μM DCFH-DA probe at 37°C for 30 min. After centrifugation, the cells were washed three times with serum-free DMEM and analyzed immediately by fluorescent microscope. For ectopic loading, cells were harvested at the indicated time points post-infection, washed twice with pre-cooled PBS, and then incubated with 1 mL of 10 μM DCFH-DA probe for 30 min at 37°C. Following centrifugation and washes with serum-free DMEM, the samples were subjected to flow cytometry analysis.

### Transfection with mRFP-GFP-LC3 adenovirus

Autophagic flux in cpBVDV/ncpBVDV-infected BT cells was monitored using the mRFP-GFP-LC3 adenoviral reporter system. This assay exploits the differential pH stability of GFP and mRFP: GFP fluorescence is quenched in the acidic environment of lysosomes, while mRFP remains stable. Thus, the autophagosomes (GFP^+^/mRFP^+^) appear as yellow puncta in merged images, whereas autolysosomes (GFP^−^/mRFP^+^) are identified as red puncta. Briefly, BT cells were seeded on glass coverslips in 12-well plates and grown to ~70% confluence. Cells were then co-infected with the Ad-mCherry-GFP-LC3B (C3011; Beyotime, China) at MOI=1 and either cpBVDV (MOI=1) or ncp BVDV (MOI = 5) for 2 h at 37°C. After infection, the medium was replaced with 500 μL of DMEM containing 2% FBS, and incubation continued for 24-48 h. LC3 puncta were finally visualized by laser confocal microscopy (FV1000, Olympus, Japan).

### IFA of viral dsRNA

We performed immunofluorescence staining to detect viral dsRNA in BT cells co-infected with the Ad-mCherry-GFP-LC3B. Cells were collected, fixed with 4% paraformaldehyde for 15 min at room temperature, and permeabilized with 0.2% Triton X-100 for 10 min. The cells were then incubated overnight at 4°C with a mouse anti-dsRNA antibody (#76651; CST, USA). Following primary antibody incubation, the cells were stained with an AF647-conjugated goat anti-mouse IgG antibody (A0473; Beyotime, China) for 30 min at 37°C. Nuclei were counterstained with DAPI for 3 min in the dark. Imaging was performed using a laser confocal microscopy (FV1000, Olympus, Japan), and the resulting images were analyzed using ImageJ software.

### Cell viability

The potential cytotoxicity of various compounds was assessed using the CCK-8 assay. BT cells were treated for 48 h with 0.5% DMSO (vehicle control), Rapa, 3-MA, MG132, CQ, BHA, 4-PBA, and jetPRIME reagent, each at their respective working concentrations. After treatment, the medium was replaced with fresh medium containing 10 μL of enhanced CCK-8 solution, and cell viability was measured according to the manufacturer’s protocol. All experiments were independently repeated three times.

### Statistical analysis

Data are expressed as mean ± SD from at least three independent experiments. Statistical analyses were performed using GraphPad Prism 8.0 software. Differences between two groups were assessed by an unpaired Student’s *t*-test, while comparisons across multiple groups were analyzed by one-way analysis of variance. Statistical significance is denoted as follows: * *P* < 0.05, ** *P* < 0.01, *** *P* < 0.001, **** *P* < 0.0001; ns, not significant.

## Data Availability

Data reported in this paper are available from the lead contact (Yigang Xu, yigangxu@zafu.edu.cn) upon reasonable request.
